# Impact of travel time to health facilities on perinatal outcomes: a systematic review with narrative synthesis and meta-analysis

**DOI:** 10.7189/jogh.16.04136

**Published:** 2026-05-15

**Authors:** Sanam Roder-DeWan, Marwa Ramadan, Paul Ouma, Alexander Manu, Emelda A Okiro, Natalie Strobel, Bibian N Robert, Emily Odipo, Peter M Macharia, Anthony Danso-Appiah, David C Goodman, Queen Dube, Gagan Gupta, Hema Magge, Karen Edmond

**Affiliations:** 1 Dartmouth Health and Dartmouth Geisel School of Medicine, Department of Community and Family Medicine: One Medical Center Drive, Lebanon, NH, USA; 2 Dartmouth Geisel School of Medicine, The Dartmouth Institute for Health Policy and Clinical Practice, Hanover, NH, USA; 3 World Bank Group, Washington, DC, USA; 4 Alexandria University, Department of Community Medicine, Alexandria, Egypt; 5 Department of Epidemiology and Disease Control, School of Public Health, University of Ghana, Legon, Accra, Ghana; 6 London School of Hygiene and Tropical Medicine, London, UK; 7 Population and Health Impact Surveillance Group, Kenya Medical Research Institute-Wellcome Trust Research Programme, Nairobi, Kenya; 8 Nuffield Department of Medicine, Centre for Tropical Medicine and Global Health, University of Oxford: Radcliffe Primary Care Building, Oxford, UK; 9 Kurongkurl Katitjin, Edith Cowan University, Perth, Western Australia, Australia; 10 Department of Public Health, Institute of Tropical Medicine, Antwerp, Belgium; 11 Centre for Evidence Synthesis and Policy, University of Ghana, Legon, Accra, Ghana; 12 Dartmouth Geisel School of Medicine, Department of Pediatrics, Hanover, NH, USA; 13 World Health Organization, Newborn and Child Health and Development Unit, Geneva, Switzerland; 14 United Nations International Children’s Emergency Fund, New York, NY, USA; 15 Brigham and Women's Hospital, Division of Global Health Equity: Boston, MA, USA; 16 Gates Foundation, Seattle, WA, USA

## Abstract

**Background:**

Evidence-based global guidance on safe travel time for small or sick newborns who require transfer to health facilities after birth is lacking. A two-hour threshold is frequently cited in low- and middle-income countries (LMICs), while 30 minutes is commonly used in high-income countries (HICs). Although these thresholds are widely referenced, their empirical basis and consistency across levels of newborn care and journey types have not been systematically examined. This study synthesises the evidence linking travel time and perinatal outcomes.

**Methods:**

We conducted a systematic review with narrative synthesis and meta-analysis to assess the impact of travel time or distance (home-to-facility or interfacility) on stillbirth, perinatal mortality, and neonatal mortality. We searched Embase, MEDLINE, and Cochrane Central Register of Controlled Trials for published studies from 2014 to 2023. Given substantial methodological heterogeneity, we used narrative synthesis as the primary analytical approach and conducted random-effects meta-analyses where studies were sufficiently comparable (≥2 with similar definitions and outcome windows), pooling effect estimates for travel time thresholds of 30 minutes, 1 hour, or 2 hours and travel distances of 5, 10, and 15 km. We assessed bias using the Newcastle-Ottawa Scale for cohort and case-control studies.

**Results:**

Of 8317 screened records, 166 were eligible for full-text review, with 37 studies meeting the inclusion criteria- All but two had low or moderate risk of bias. Most studies (n = 26) came from LMICs and documented higher perinatal survival with shorter journeys. Studies from HICs demonstrated lower out-of-hospital birth, lower morbidity, and lower mortality with shorter journeys though associations were weaker. Across the narrative synthesis, shorter travel times were consistently associated with better outcomes. Exploratory pooling suggested a greater than 3-fold higher odds of survival for interfacility journeys under 30 minutes (odds ratio (OR) = 3.25; 95% confidence interval (CI) = 1.90–5.57) and over 2-fold higher odds of survival for journeys from any location to hospitals at all thresholds (OR = 2.06; 95% CI = 1.60–2.65 for 2 hours; OR = 2.20; 95% CI = 1.46–3.33 for 1 hour; OR = 1.92; 95% CI = 1.10–3.34 for 30 minutes), though prediction intervals were wide, reflecting methodological and contextual diversity.

**Conclusions:**

We found that shorter journeys were associated with better perinatal outcomes, with the highest survival rates observed for journeys under 30 minutes to the hospital. Due to substantial contextual and methodological heterogeneity, pooled estimates should be interpreted as illustrative, rather than definitive. A travel time norm of 30 minutes or one hour is preferable to the two-hour threshold currently used in LMICs. To safeguard perinatal survival rates, any travel-time standard should be balanced with corresponding quality of care standards.

**Registration:**

PROSPERO: CRD42023460423.

The newborn period remains the riskiest time of life for children worldwide. Although significant progress has been made in reducing newborn mortality, approximately 2.3 million children died within the first month of life in 2023 alone [1]. The World Health Organization (WHO) estimates that 80% of neonatal deaths occur in those born too early or with a low birthweight [2]. Another 1.9 million babies are stillborn, 40% of whom die during labour [3]. Perinatal deaths are inequitably distributed across the globe and are slower to decline in the highest burden settings. A baby born in the highest mortality country is 65 times more likely to die in the neonatal period (within one month of birth) than one born in the lowest mortality country [1,3].

Small and sick newborns (SSNs) often need urgent specialised care to survive, which is not available at every birth. High-quality prenatal care can identify women at higher risk of giving birth to an SSN and direct them to give birth in higher-level facilities. However, some complications are difficult to predict, and many babies who need specialised care are born at home or in a facility lacking these services. Across settings, approximately 30% of children in low- and middle-income countries (LMICs) are born outside of a hospital [4–6].

WHO and the United Nations International Children’s Emergency Fund (UNICEF) identify three levels of newborn care [7]. Level I is for babies who need only essential newborn care. Level II specialised neonatal care is usually available in first-level hospitals and higher and includes care for common complications and the ability to refer to Level III intensive care. In the absence of universal and immediate access to specialised care – which is needed by more than 30 million newborns globally each year [7] – travel time to an appropriate facility (or ‘safe journey time’) becomes a key determinant of survival and a health system imperative. The fourth global Every Newborn Action Plan (ENAP) target is to ensure at least one Level II SSN care unit in every district (or equivalent) in every country [8].

A two-hour threshold is commonly cited in the global literature as a safe journey time for maternal and newborn health services, but the evidence base for it is lacking. The time threshold originates from a presentation given at the International Safe Motherhood Conference (1987), estimating the average time-to-death for common obstetric complications [9]. It was then referenced in guidelines for the monitoring of emergency obstetric care [9,10], adopted by the global surgical community [11], incorporated into the WHO global reference list of 100 core health indicators [12], and referenced in the most recent efforts to improve outcomes for mothers and babies globally [13]. The level of newborn care that should be available is also often not specified. More recently, there have been calls to rethink travel time thresholds and the two-hour target in LMICs [5,14].

Though controversial, a 30-minute threshold is frequently used in high-income countries (HICs) and is derived from human and animal studies estimating the interval between the onset of foetal distress and poor neonatal outcomes [15–18]. It is commonly defined as the obstetric decision-to-incision interval for emergency caesarean section. Despite concerns over the feasibility of achieving the threshold and its applicability to all conditions, it has been incorporated into clinical guidelines [19–21] and is used by planners and scholars. Of note, there is no standard measurement approach for spatial accessibility in healthcare, leading to variability in reporting and challenges in comparing journeys [22].

While both thresholds are frequently used, their empirical basis and applicability across journey types and levels of care remain uncertain. Here, we synthesise the available evidence on the relationship between travel time to hospitals and perinatal outcomes, addressing this long-standing evidence gap.

## METHODS

We used standard Cochrane methods [23] for observational systematic reviews to evaluate the impact of proximity to health facilities on stillbirth, perinatal and neonatal mortality. We preregistered the study protocol on PROSPERO [24] and followed the Preferred Reporting Items for Systematic Reviews and Meta-Analyses-Protocol (PRISMA-P) guidelines [25,26].

While the original protocol anticipated a full meta-analysis, initial screening revealed substantial heterogeneity in study design, exposure measurement, facility classification, and outcome definitions. To preserve methodological rigour and transparency, we adopted a narrative synthesis approach consistent with Synthesis Without Meta-analysis (SWiM) guidance [27], supplemented by meta-analyses in cases where study definitions and methods were sufficiently comparable.

### Study design and population

We included population-based observational studies (*e.g.* cohort, cross-sectional, and case-control studies) and excluded case reports and abstracts. Studies had to include infants younger than 28 days of age or stillbirths. Both community-based and facility-based studies were eligible, including those examining either home-to-facility journeys or interfacility transfers to higher-level care.

### Exposures

We defined the exposure as travel time or distance from the point of origin to a health facility. We included all health facility types and classified them as described in the original study. The point of origin included any place of birth, such as home or lower-level health facilities. Studies reported travel time in minutes or hours, which was derived from either self-reported data or geospatial models, incorporating transport mode and factors affecting travel, such as terrain.

We defined distance as the physical space between the point of origin and the facility, measured as straight-line or road-network distance in kilometres or miles. We focused on thresholds commonly used to define limited geographic access: ≤30 minutes, ≤1 hour, and ≤2 hours; ≤5 km, ≤10 km, and ≤15 km.

We classified individuals who reached care within the specified threshold as the reference group and those who exceeded it as the exposed group. Because studies used heterogeneous proximity definitions, we harmonised exposures post-extraction. When studies reported non-standard or uneven time bands, we used raw exposure data from study tables to recompute comparable thresholds wherever possible. We analysed continuous travel-time and distance measures separately.

### Outcomes

Primary outcomes were all-cause neonatal mortality (deaths among live births during the first 28 days of life), stillbirth (death after 28 weeks of pregnancy, before or during birth), perinatal mortality (the sum of the number of perinatal deaths (stillbirths and early neonatal deaths ≤7 days)) divided by the number of pregnancies of seven or more months’ duration (all live births plus stillbirths). Secondary outcomes were major cause-specific mortality for stillbirths, neonatal mortality, and perinatal mortality. We conducted the meta-analysis using mortality data; however, for ease of interpretation, the results are presented as odds of survival (*i.e.* the inverse of mortality).

### Search strategy

We searched MEDLINE, Embase, the Cochrane Central Register of Controlled Trials, and protocol registries (Figure S1 in **Online Supplementary Document**). We used search terms for the population, travel time or distance, health facilities, and the outcomes. We included English-language studies published between January 2014 and September 2023. The initial protocol specified a narrower window (2019–2023); however, after screening revealed too few eligible studies, we extended the range to 2014–2023. This 10-year period corresponds to the ‘modern’ era of SSN care, when continuous positive airway pressure was explicitly consolidated in global newborn norms as a component of transitional care from Level II to Level III inpatient care and incorporated into ENAP’s fourth coverage target and the WHO standards for improving the quality of care for SSNs in health facilities [8].

### Study selection and data extraction

Two reviewers independently screened titles and abstracts and assessed the full texts for eligibility. Disagreements were resolved through discussion until a consensus was reached. Then, we developed and pretested a data extraction form, in which two reviewers independently extracted data from each record after verification. Discrepancies were also resolved through discussion until consensus was reached or, if necessary, by consulting another author. We extracted data on title, authors, publication year, country, dates of data collection and last assessment, gestational age, sex, birthweight, ethnicity, travel-time data as reported by the authors, mode of travel, proxies for travel time (distance or geospatial mapping), and methods used to generate travel time estimates. We documented detailed data on health facilities, outcomes, any known confounders used in the study, the effects of our exposure of interest and any funding sources or conflicts of interest. We used the Covidence systematic review software [28] to manage all stages between de-duplication and extraction.

### Assessment of methodological quality and evidence

Two teams of reviewers independently assessed the risk of bias of included studies using the Newcastle-Ottawa Scale (NOS) for cohort and case-control studies [29] and a piloted, adapted quality assessment for cross-sectional studies [30]. At least two reviewers evaluated each study, with discrepancies resolved through consultation with a third reviewer. Another reviewer (MR) corroborated a randomly selected sample (10%) of assessments.

Given the small number of included studies contributing to the pooled analyses, we did not conduct formal publication bias tests (funnel plots and Egger’s test) [31]. We considered potential publication bias narratively by examining study characteristics, funding sources, and selective outcome reporting.

### Data synthesis

First, we summarised all included studies narratively, emphasising the direction, magnitude, and consistency of associations between proximity and perinatal outcomes. For dichotomous outcomes, we summarised results using risk ratios (RRs), and used odds ratios (ORs) where RR calculations were not feasible, which approximate RR in studies with rare outcomes. For continuous outcomes, we used standardised mean differences to account for variability in measurement methods and scales across studies; otherwise, we used mean (x̄) difference with standard deviation (SD). We reported all effect estimates with their 95% confidence intervals (CIs). Where data permitted quantitative pooling, we computed effect sizes separately for travel time and travel distance. For travel time, we pooled categorical thresholds (≤30 minutes, ≤1 hour, ≤2 hours) independently from continuous measures; for distance, we performed pooling at 5, 10, and 15 km thresholds. When studies reported non-standard exposure bands, we used raw exposure data to recompute and align thresholds before pooling. We retained studies that could not be standardised in the narrative synthesis only. When available, we extracted study-level adjusted effect sizes to generate pooled estimates; otherwise, we used raw data. We handled missing data according to Cochrane-recommended imputation methods, with details specified in individual study assessments.

We assessed effects and conducted a narrative synthesis across these variables: outcome; facility type and level; transfer type (interfacility *vs.* home-facility); country wealth group; region; neonatal mortality band (neonatal mortality rate <40 and ≥40); mode of travel (motorised, bicycle, walk, hybrid); type of proximity measure; proximity methods (cost distance, straight line, others). We conducted exploratory meta-analysis only where methodological and definitional consistency was adequate (≥2 comparable studies). For all other subsets, we summarised results narratively, integrating both direction and strength of association to describe overall patterns in the evidence.

We conducted statistical analyses using the metafor package in *R*, version 4.3.3 (R Core Team, Vienna, Austria) and Stata, version 16. 1 (StataCorp LLC, College Station, TX, USA) [32].

## RESULTS

**Figure 1:**
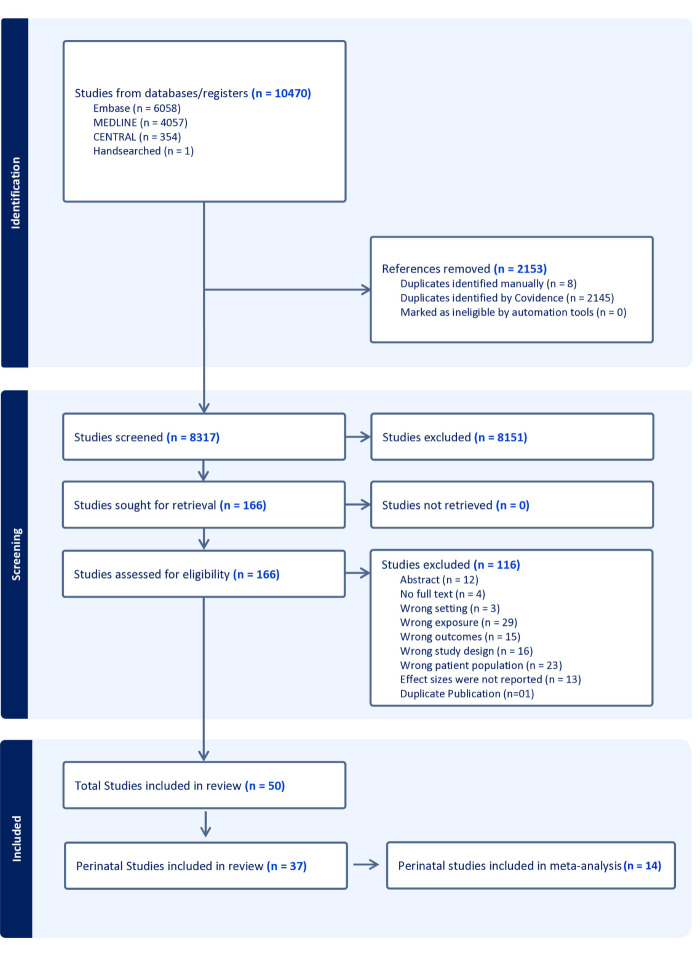
PRISMA flow diagram.

The search generated 8317 records, which we screened for inclusion criteria (**Figure 1****).** Of these, 166 were eligible for full-text review, and 116 were excluded at this stage. Finally, we included 37 studies examining perinatal mortality (**Table 1**) [33–59,61–69]. Given the heterogeneity in design, exposures, and outcome definitions, we synthesised findings narratively and calculated pooled estimates where comparable data were available. Among these, we included 14 studies in the meta-analysis, which provided sufficient data for estimating effect sizes (**Table 2**) [33,37,41,43–45,47,52,54,56,57,59,61].

**Table 1:** Included studies

			Exposure			
**Author, year, reference**	**Country**	**Study design**	**Place of birth**	**Main exposure**	**Method used**	**Time or distance thresholds**
**Low-and middle-income country studies**						
Bokade *et al.*, 2018 [33]	India	Cohort	Tertiary (teaching hospital)	Distance	Straight line distance	Mean distance among surviving and non-surviving neonates
Caviglia *et al.*, 2021 [34]	Sierra Leone	Cohort	Secondary	Travel time	Operational ambulance time	2 hours
El Hiyani *et al.*, 2023 [35]	Morocco	Case control	Secondary (regional hospital)	Distance	Straight line distance	5km, 5–50km, >50
Gizaw *et al.*, 2014 [36]	Ethiopia	Cohort	Secondary	Distance	Straight line distance	5 and 10 km
Jarde *et al.*, 2021 [37]	Gambia	Cohort	Primary	Distance	Straight line distance	Distance in km
Khan *et al.*, 2022 [38]	Bangladesh	Cross-sectional	All levels	Distance	Straight line distance	Continuous distance in km
Kibret *et al.*, 2023 [39]	Ethiopia	Cross-sectional	All levels	Distance	Network distance (offline GIS software)	Distance in km as continuous variable (for every 10 km) 10km time bands
Macharia *et al.*, 2023 [40]	Tanzania	Cross-sectional	Secondary (hospitals)	Motorised travel time	Least cost path analysis (time)	Continuous time
McDiehl *et al.*, 2021 [41]	Uganda	Cohort	Secondary (regional hospital)	Distance	Straight line distance	Distance in km
McKinnon *et al.*, 2014 [42]	Ethiopia	Cross-sectional	All	Distance	Straight line distance	Cut-off 5km, 10 km can be computed
Milton *et al.*, 2022 [43]	Nigeria	Cross-sectional	Tertiary (national teaching hospital)	Distance	Straight line distance	distance reported as cut offs <10 km, 10–30, 31–50, 51–100, more than 100
Narang *et al.*, 2013 [44]	India	Cross-sectional	Tertiary and secondary (medical college)	Referral time – motorised	Measured transport time	Time (continuous & Categorical), <1 hours, 1–2 hour, 2–4, 4–6 hours, more than 6 hours
Niyitegeka *et al.*, 2017 [45]	Rwanda	Cross-sectional	Secondary (caesarean services)	Referral time – motorised	Measured transport time	90 minutes
Ouma *et al.*, 2022 [46]	Kenya	Cross-sectional	Secondary (caesarean services)	Motorised travel time	Least cost path analysis (time)	2 hours
Rosario *et al.*, 2019 [47]	Angola	Cross-sectional	All levels	Distance	Network distance (offline GIS software)	<2 km, 2–5 km, 6–10 km, 11–15 km, >15 Km
Sarrassat *et al.*, 2019 [48]	Burkina Faso	Cross-sectional	All levels	Distance	Straight line distance	<2, 2–4,4–7, more than 7
Singh *et al.*, 2021 [49]	India	Cross-sectional	Tertiary	Referral time – motorised	Measured transport time	2 hours
Stevens *et al.*, 2016 [50]	South Africa	Cross-sectional	Tertiary	Time and distance	N/A	Continuous time and distance
Tayler-Smith *et al.*, 2013 [51]	Burundi	Cross-sectional	Secondary (CEMONC)	Referral time – motorised	Measured transport time	3 hours
Tette *et al.*, 2020 [52]	Ghana	Cross-sectional	Secondary (regional hospital and other hospitals)	Motorised travel time	Network distance and time (offline GIS software)	Distance and time: expressed as median time and IQR among those who died and survivors
Ullrich *et al.*, 2020 [53]	Uganda	Cross-sectional	Tertiary and secondary	Distance	Straight line distance	Distance travelled in km with 25 cut-off /9km) from 0 till >200
Van Duinen *et al.*, 2020 [54]	Sierra Leone	Cohort	Secondary (caesarean services)	Reported	Patient reported	20 minutes, 30 minutes, 2 hours
Viswanath *et al.*, 2015 [55]	India	Case control	All	Travel time	N/A	2 hours
Wariri *et al.*, 2021 [56]	Nigeria	Case control	Tertiary not reported to have intensive care	Motorised travel time	Least cost path analysis (time	Time, divided into Time to nearest facility categories 5 minutes and below, 16–10 minutes, 11–15 minutes, 16–20 minutes, 21 minutes and above. Time-to-the-nearest-hospital divided into 15minutes and below, 16–30 minutes, 31–45 minutes, 46–60 minutes, 60 minutes and above
Yeshaneh *et al.*, 2021 [57]	Ethiopia	Cohort	Tertiary (specialised hospital)	Motorised travel time	Measured transport time	30 minutes, 60 minutes, 90 minutes, 120 minutes; Time
**High-income country studies**						
Bergh *et al.*, 2020 [58]	USA	Cohort	Tertiary	Distance	Straight line distance	<250 miles, 250–499 miles, ≥500 miles
Combier *et al.*, 2013 [59]	France	Cross-sectional	Secondary (maternity units)	Motorised travel time	Network travel time (offline GIS software)	<15 min, 15–30 minutes, 30 minutes-45 minutes, >45 minutes
Combier *et al.*, 2020 [60]	France	Cohort	Secondary (maternity units)	Distance	Network distance (offline GIS software)	0–15, 16–20, 31–45, 46–90 km
Featherstone *et al.*, 2016 [61]	USA	Cross-sectional	Secondary	Motorised travel time	Network time (offline GIS software)	30 minutes, and one hour (computed)
Lee *et al.*, 2022 [62]	Korea	Cross-sectional	Tertiary	Motorised travel time	Network travel time (offline GIS software)	60 minutes
Paranjothy *et al.*, 2014 [63]	UK (Wales)	Cohort	Secondary	Motorised travel time	Network travel time (Google)	15-minute time bands
Patel *et al.*, 2022 [64]	USA	Cohort	Secondary (surgical centre)	Distance	Not clear	<5 miles, 5–19 miles, 20–100 miles, and >100 miles
Pilkington *et al.*, 2014 [65]	France	Cohort	Secondary (maternity Units)	Distance	Network distance (offline GIS software)	5km, and 15 km (computed)
Purkey *et al.*, 2022 [66]	USA	Cohort	Secondary (birth hospitals)	Distance	Straight line distance	11 miles
Singh *et al.*, 2021 [67]	USA	Other	Tertiary	Distance	Road network and helicopter distance	Continuous distance in miles
Swartz *et al.*, 2017 [68]	USA	Cross-sectional	Tertiary	Referral time – motorised	Network distance (Google)	11–90 miles, >90 miles
**Multi-country studies**						
Karra *et al.*, 2017 [69]	Multi-country	Cross-sectional	All	Distance	Perceived distance	Distance, 2km, 3km, 5km, 10km

CEMONC – comprehensive emergency obstetric and newborn care, GIS – geospatial information systems, IQR – interquartile range, km – kilometres, N/A – not available

**Table 2:** Summary of outcomes in included studies

Author, year, reference	Outcome category	Effect measure and size	Reference	Adjusted	Covariates adjusted for	Summary of findings	Included in pooled analysis
**Low- and middle-income country studies**							
Bokade *et al.*, 2018 [33]	NMR	Mean distance non-survivors 94.13 km (SD = 77.58) vs survivors 80.38 km (SD = 74.96), *P* = 0.006	Continuous referral distance (km); comparison of mean distance travelled by neonatal deaths vs survivors	No	Not applicable	Longer referral distance was associated with higher neonatal mortality. Non-survivors travelled farther than survivors and the difference in mean distance was statistically significant. Unadjusted descriptive comparison.	Yes (SMD)
Caviglia *et al.*, 2021 [34]	Perinatal mortality	Risk of perinatal death: 60 min = 16%, 120 min = 18%, 180 min = 25%. *P*-value not reported for effect estimates (modified Poisson regression used)	Pre-hospital transport time in minutes (continuous); no explicit categorical reference *vs.* contrast reported	Not clearly reported (model adjusted but numeric estimates not given)	Not reported	Perinatal mortality increased progressively with longer transport time. The risk rose from 16% at 60 minutes to 25% at 180 minutes. Modified Poisson regression with natural cubic splines was used but final adjusted risk ratios were not provided. Based on 1717 referrals.	
El Hiyani *et al.*, 2023 [35]	NMR	aOR = 3.588 (95% CI = 1.952–6.594), *P* < 0.001 for 5–50 km; aOR = 0.295 (95% CI = 0.138–0.633), P < 0.001 for >50 km	Distance to referral centre: <5 km (reference) = 5–50 km and >50 km	Yes	Parity, number of prenatal visits, ultrasound exam, referral, mode of birth, low birthweight, 5-minute Apgar score, gestational age	Neonates whose referral centre was 5–50 km from the hospital had about 3.6 times higher odds of neonatal death compared with those <5 km. Distances >50 km were associated with lower odds of death. Based on 760 infants.	
Gizaw *et al.*, 2014 [36]	NMR	aOR = 1.5 (95% CI = 1.1–2.0) for 5–9 km; aOR = 1.3 (95% CI = 0.9–1.8) for ≥10 km; crude OR = 2.0 (95% CI = 1.7–2.5) for 5–9 km; crude OR = 2.2 (95% CI = 1.8–2.6) for ≥10 km. *P*-values not reported.	Distance to hospital: <5 km (reference) = 5–9 km, ≥10 km	Yes	Sex, residential area, water source, house ownership, religion, type of roof, distance to hospital	Neonatal mortality increased with greater distance to hospital. Adjusted incidence rate ratios showed elevated risk at 5–9 km and a smaller, non-significant elevation at ≥10 km. Study included 226 neonatal deaths in Poisson regression.	
Jarde *et al.*, 2021 [37]	NMR	HR = 0.96 (95% CI = 0.78–1.19), *P* = 0.735	Distance to PHC facility (continuous); no explicit reference threshold	No	Not applicable	Distance to the nearest PHC facility was not associated with neonatal mortality. The effect was null and non-significant. Median travel distance for neonates was 0.5 km (IQR 0–1.5 km).	Yes (SMD)
Khan *et al.*, 2022 [38]	NMR	aOR = 1.20 per 1 km increase (95% CI = 1.01–1.45), *P* < 0.01; crude OR = 1.28 (95% CI = 1.03–1.55)	Distance to nearest child-health-service facility (continuous, per 1 km); no explicit categorical reference	Yes	Maternal age at birth, maternal education, maternal employment, partner education, sex of household head, parity, media exposure, wealth quintile, residence, region	Each additional kilometre between the mother’s home and the nearest facility was associated with a 20% increase in the odds of neonatal death. The effect remained significant after adjustment. Sample: 8,759 births, 189 neonatal deaths.	
Kibret *et al.*, 2023 [39]	NMR	aOR = 1.33 per 10 km increase (95% CI = 1.06–1.67), *P* = 0.01. Authors also report about 6%, 15% and 22% higher odds per 2, 5 and 7 km increments (no explicit CIs).	Distance to nearest health facility (continuous, per 10 km); no categorical reference *vs.* contrast	Yes	ANC care, facility delivery, postnatal counselling, counselling on danger signs, term pregnancy, cord care practices, gender of child, residence type, type of pregnancy	For every 10 km increase in distance to the nearest health facility, the odds of neonatal mortality increased by 33%. Distance was also linked to reduced use of maternal services.	
Macharia *et al.*, 2023 [40]	NMR and perinatal mortality	Neonatal mortality OR = 0.92 (95% CI = 0.75–1.13), *P* = 0.425; perinatal mortality OR = 0.97 (95% CI = 0.86–1.10), *P* = 0.675	Modelled travel time to hospital (continuous hours); no categorical reference threshold	No	Not applicable	Travel time to hospital was not associated with neonatal or perinatal mortality. ORs were close to 1 with wide CIs and non-significant p-values in this nationally representative DHS-linked dataset (N = 8,915).	
McDiehl *et al.*, 2021 [41]	Stillbirth	aOR = 1.0 (95% CI = 1.0–1.0) for distance travelled, *P* = 0.08	Distance from home to hospital (continuous, km); no explicit reference threshold	Yes	Age, parity, distance travelled, referral status, syphilis infection during pregnancy	Distance to hospital was not significantly associated with stillbirth. ANC attendance ≥4 visits significantly reduced stillbirth, but distance contributed no measurable effect despite inclusion in the model.	Yes (SMD)
McKinnon *et al.*, 2014 [42]	Early NMR	Adjusted marginal effect for >80 km from nearest CEmONC: +14.4 deaths per 1000 live births (95% CI = 0.1–28.7). *P*-value not explicitly reported.	Distance to nearest CEmONC facility: ≤10 km (reference) = >80 km (plus intermediate categories)	Yes	Poverty, maternal age, sex of child, birth interval and order, multiple birth, regional residence, maternal and paternal education, women’s autonomy	Living >80 km from a CEmONC facility was associated with significantly higher early neonatal mortality (+14.4 per 1000 live births). Lower distance categories showed increasing but mostly non-significant marginal effects.	
Milton *et al.*, 2022 [43]	Stillbirth	aOR = 1.52 (95% CI = 1.08–2.12), *P* = 0.015 for 10–30 km; aOR = 3.64 (95% CI = 1.93–6.85), *P* < 0.001 for 31–50 km; aOR = 3.39 (95% CI = 1.25–9.21), *P* = 0.017 for 51–100 km; aOR = 4.44 (95% CI = 1.31–15.05), *P* = 0.017 for >100 km *vs..* <10 km	Distance from home to hospital: <10 km (reference) = 10–30, 31–50, 51–100, >100 km	Yes	Mother’s age, household monthly income, employment status, education level, type of residence, water source, household water facilities, self-perceived nutritional status, medications and supplements, health workers, health conditions, pregnancy history, labour duration, delivery, antepartum haemorrhage	Greater distance from home to hospital was strongly associated with higher odds of stillbirth. Odds increased progressively with distance, showing a dose–response pattern.	Yes (10 Km) Distance
Narang *et al.*, 2013 [44]	NMR	Multivariable: OR = 5.58 (95% CI = 1.41–22.01) for transport time >1 hour *vs*. ≤1 hour, *P* = 0.01. Univariable by category: 1–2 hours OR = 2.52 (95% CI = 0.86–7.34), *P* = 0.06; 2–4 hours OR = 7.36 (95% CI = 3.03–18.27), *P* < 0.01; 4–6 hours OR = 14.31 (95% CI = 5.24–40.08), *P* < 0.01; >6 hours OR = 81.75 (95% CI = 21.39–347.0), *P* < 0.01.	Transport time to hospital: ≤1 h (reference) = 1–2, 2–4, 4–6, >6 h	Yes	Multivariate model identified birthweight <1 kg and transport time >1 h as independent predictors; other covariates not fully detailed	Transport time >1 h was significantly associated with neonatal mortality. Univariable estimates suggested a steep increase in mortality with longer transport times, with ORs rising to >80 for >6 h. Longer transport time and very low birthweight jointly drove much higher risk of death.	Yes (1 Hr) Interfacility
Niyitegeka *et al.*, 2017 [45]	NMR (death or low Apgar)	aOR = 3.02 (95% CI = 0.84–10.84), *P* = 0.09 for 30–<60 min; aOR = 4.31 (95% CI = 1.02–18.29), *P* = 0.05 for 60–<90 min; aOR = 5.12 (95% CI = 1.30–20.21), *P* = 0.02 for ≥90 min *vs.* <30 min	Travel time from health centre to district hospital: <30 min, including same-compound (reference) = 30–<60, 60–<90, ≥90 min	Yes	Models adjusted for district hospital and other potential confounders; separate multivariate regressions for each delay predictor	Longer referral travel time remained significantly associated with unfavourable neonatal outcome (death or low Apgar). Compared with <30 min, travel time 60–<90 min and ≥90 min showed a strong graded increase in odds.	Yes (30 min) Interfacility
Ouma *et al.*, 2022 [46]	NMR	Regression coefficient −0.235 (95% CI = −0.463 to −0.007), *P* = 0.049	Percentage of population within 2 hours of VLBW hospital, continuous	Yes	Wealth, health workforce density, maternal education	A 1-percentage-point increase in population living within 2 hours of a very-low-birthweight hospital was associated with a 0.235-point reduction in neonatal mortality. Effect estimated from multivariable linear regression.	
Rosário *et al.*, 2019 [47]	Stillbirth	aORs by distance *vs.* >15 km: <2 km 0.64 (95% CI = 0.27–1.53), *P* ≈ 0.32; 2–5 km 0.80 (95% CI = 0.30–2.15), *P* ≈ 0.66; 6–10 km 1.93 (95% CI = 0.67–5.55), *P* ≈ 0.22; 11–15 km 1.44 (95% CI = 0.52–4.02), *P* ≈ 0.49	Distance to facility: >15 km (reference) = <2, 2–5, 6–10, 11–15 km	Yes	ANC attendance, place of residence, place of delivery, education, women’s age	Proximity to a health facility reduced stillbirth odds in bivariate analysis (significant only for <2 km). In adjusted models none of the distance categories were statistically significant, though shorter distances tended to be protective and longer distances harmful.	Yes (5km, 10 Km, 15 km) Distance
Sarrassat *et al.*, 2019 [48]	NMR	RRs *vs.* <2 km: 2–4 km RR = 0.94 (95% CI = 0.82–1.08), *P* = 0.397; 4–7 km RR = 1.09 (95% CI = 0.95–1.25), *P* = 0.219; >7 km RR = 1.19 (95% CI = 1.03–1.38), *P* = 0.021; 10–20 km RR = 1.03 (95% CI = 0.92–1.17), *P* = 0.575; >20 km RR = 1.08 (95% CI = 0.90–1.28), *P* = 0.404. *P*-trend for distance = 0.014.	Distance to nearest facility: <2 km (reference) = 2–4, 4–7, >7, 10–20, >20 km	Yes	Household wealth quintile, mother’s age, child’s gender and age group, ethnicity, religion, maternal education, marital status, duration in village, birth order, birth interval	After adjustment, neonatal mortality increased with distance and was significantly higher beyond 7 km. A trend test showed increasing neonatal mortality with increasing distance. No association was found for post-neonatal mortality.	
Singh *et al.*, 2021 [49]	NMR	Mean transfer time 17.0 hours (95% CI = 10.7–23.21), *P* = 0.123; mean travel distance 239.2 km (95% CI = 193.4–285.0), *P* = 0.27	Continuous transfer time (hours) and distance (km); no explicit reference category	Yes (but distance/time not retained as predictors)	Factors significant at p < 0.150 were adjusted in the model, though transfer time and distance were not significant	Transfer time and travel distance were not significant predictors of neonatal mortality in gastroschisis patients. Travel time did not influence survival in this cohort.	
Stevens *et al.*, 2016 [50]	NMR	Same estimates reported as Singh *et al.* (transfer time mean 17.0 hours, 95% CI = 10.7–23.21, *P* = 0.123; travel distance mean 239.2 km, 95% CI = 193.4–285.0, *P* = 0.27)	Continuous transfer time (hours) and distance (km); no explicit reference category	Yes (multivariate regression, but distance/time not retained as predictors)	Factors significant at p < 0.150 were adjusted, distance/time were not significant	Transfer time and travel distance were not significant predictors of neonatal mortality.	
Tayler-Smith *et al.*, 2013 [51]	Stillbirth and NMR within 24 hours	OR = 1.9 (95% CI = 1.1–3.2), *P* = 0.02 for referral time ≥3 hours *vs.* <3 hours	Referral time: <3 hours (reference) = ≥3 hours	No (crude)	None reported	Referral time ≥3 hours was associated with almost double the odds of early neonatal death. Stillbirths were combined with neonatal deaths in the analysis.	
Tette *et al.*, 2020 [52]	NMR	Descriptive only. Median distance (died) 18.27 km (range 2.69–75.66) *vs.* survived 18.27 km (0.93–91.15); median distance died (second dataset) 21.17 km; time travelled 60 *vs.* 90 minutes. No *P*-values.	Distance in km and travel time in minutes; no defined reference threshold	No	None	No statistical relationship was modelled or tested. Authors only compared medians and proportions. Mortality was not analytically linked to distance or time.	Yes (SMD)
Ullrich *et al.*, 2020 [53]	NMR	Chi-square: χ^2^(8) = 19.760, *P* = 0.011 (significant) in full cohort; χ^2^(8) = 4.7, *P* = 0.794 (non-significant) in congenital anomalies subgroup.	Distance categories 0–25, 26–50, 51–75, 76–100, 101–125, 126–150, 151–175, 176–200, >200 km; no specific numeric reference	No	None (descriptive chi-square only)	Distance travelled was significantly associated with mortality in the full cohort, with mortality differing across distance groups. In the congenital anomalies subgroup the association was not significant. No OR or RR was calculated.	
Van Duinen *et al.*, 2020 [54]	Perinatal mortality	Perinatal mortality per 1000 births: reported time ≤2 hours *vs.* >2 hours 193 *vs.* 308, *P* < 0.001; modelled time (Model I) 209 *vs.* 344, *P* = 0.003; modelled time (Model II) 181 *vs.* 319, *P* < 0.001.	Travel time ≤2 hours (reference) = >2 hours (reported and modelled)	No (univariable threshold analyses)	None reported	Perinatal mortality was significantly higher among women whose reported or modelled travel time exceeded 2 hours. Threshold analyses consistently showed higher mortality with longer travel times.	Yes (2 hours) home to facility
Viswanath *et al.*, 2015 [55]	Perinatal mortality	aOR = 2.51 (95% CI = 1.08–6.73) for >2 hours vs ≤2 hours, *P* not reported.	Time to reach nearest health facility: ≤2 hours (reference) = >2 hours	Yes	Maternal age, education, parity, gestational age, ANC visits, tetanus immunisation, place of delivery	Time to reach the nearest health facility >2 hours was significantly associated with higher perinatal mortality. Regression adjusted for maternal and pregnancy characteristics.	
Wariri *et al.*, 2021 [56]	Stillbirth	Travel time to nearest facility (≤5 min reference): 6–10 min aOR = 1.8 (95% CI = 0.6–5.5), *P* = 0.277; 11–15 min aOR = 5.1 (95% CI = 0.9–28.0), *P* = 0.059; 16–20 min aOR = 0.1 (95% CI = 0.0–5.2), *P* = 0.275; ≥21 min aOR = 0.7 (95% CI = 0.3–2.0), *P* = 0.504. Travel time to FTHG (≤15 min reference): 16–30 min aOR = 0.9 (95% CI = 0.3–3.4), *P* = 0.947; 31–45 min aOR = 0.5 (95% CI = 0.1–3.5), *P* = 0.485; 46–60 min aOR = 1.2 (95% CI = 0.3–4.2), *P* = 0.822; ≥60 min aOR = 12.2 (95% CI = 1.8–24.3), *P* = 0.011.	Nearest facility: travel time ≤5 min (reference) = 6–10, 11–15, 16–20, ≥21 min. FTHG: ≤15 min (reference) = 16–30, 31–45, 46–60, ≥60 min	Yes	Mother’s age, education, occupation, parity, booking status, referral, mode of transport to FTHG	After adjustment the only significant predictor of stillbirth was travel time ≥60 minutes to FTHG, with about 12-fold higher odds than ≤15 minutes. Other categories were not significant. Sample size 318 women.	Yes (SMD) & (30 min, 1 HRs, 2 Hrs) Home to facility
Yeshaneh *et al.*, 2021 [57]	NMR	aOR = 1.54 (95% CI = 0.55–4.32) for 30–60 min; aOR = 2.15 (95% CI = 0.85–5.41) for 60–120 min; aOR = 3.8 (95% CI = 1.65–9.14) for ≥120 min *vs.* <30 min; *P* not reported for first two categories, *P* < 0.05 for ≥120 min	Travel time to referral hospital: <30 min (reference) = 30–60, 60–120, ≥120 min	Yes	Place of delivery, mode of delivery, type of pregnancy, gestational age, hypothermia at admission, hypoglycaemia at admission, oxygen desaturation <90%, prolonged capillary refill time, admission weight, age at admission, vital signs during transport, ambulance transport, residence	Neonates who travelled ≥120 minutes to reach referral hospitals had 3.8 times higher odds of death compared with those travelling <30 minutes. Travel time 30–60 and 60–120 minutes showed elevated but non-significant odds.	Yes (30 min & 1 Hr, 2 Hrs) Interfacility
**High-income country studies**							
Bergh *et al.*, 2020 [58]	NMR	Group 1 <250 miles (reference): *β* 0.00094 (SE = 0.00078), OR ≈ 1.00; Group 2 250–499 miles: *β* −0.17393 (SE = 0.32539), OR ≈ 1.03 (95% CI = 0.60–1.78), *P* = 0.593; Group 3 ≥500 miles: *β* −0.68547 (SE = 0.64993), OR ≈ 0.99 (95% CI = 0.55–1.79), *P* = 0.292. Overall *P* = 0.230 for distance as continuous.	Travel distance to fetal centre: <250 miles (reference) = 250–499, ≥500 miles, plus continuous distance	No	None (unadjusted logistic regression)	Distance travelled to the fetal centre did not predict composite neonatal morbidity. All ORs were close to 1 with wide CIs, indicating no meaningful association.	
Combier *et al.*, 2013 [59]	Stillbirth and perinatal mortality	Stillbirths *vs.* ≤15 min: aOR = 1.16 (95% CI = 0.96–1.40), *P* = 0.12 for 16–30 min; aOR = 1.31 (95% CI = 0.89–1.93), *P* = 0.17 for 31–45 min; aOR = 1.90 (95% CI = 0.70–5.15), *P* = 0.21 for ≥46 min. Perinatal mortality *vs.* ≤15 min: aOR = 1.08 (95% CI = 0.90–1.29), *P* = 0.40 for 16–30 min; aOR = 1.18 (95% CI = 0.86–1.62), *P* = 0.31 for 31–45 min; aOR = 1.85 (95% CI = 0.66–5.19), *P* = 0.25 for ≥46 min.	Travel time to maternity unit: ≤15 minutes (reference) = 16–30, 31–45, ≥46 min	Yes	Study period, maternal individual characteristics, contextual characteristics of residence	The study identified a positive gradient between increasing travel time and the risk of stillbirth and perinatal mortality. Most estimates were not statistically significant, but highest travel times showed the largest relative risks.	Yes (30 min) home to facility
Combier *et al.*, 2020 [60]	Stillbirth, NMR and neonatal morbidity	Neonatal mortality *vs.* 0–15 km: 16–30 km aRR = 1.0 (95% CI = 0.9–1.1), 31–45 km aRR = 1.0 (95% CI = 0.9–1.5), 46–90 km aRR = 0.9 (95% CI = 0.7–1.9); stillbirth *vs.* 0–15 km: 16–30 km aRR = 1.0 (95% CI = 0.9–1.0), 31–45 km aRR = 1.0 (95% CI = 0.9–1.1), 46–90 km aRR = 0.9 (95% CI = 0.7–1.2); neonatal hospitalisation *vs.* 0–15 km: 16–30 km aRR = 1.1 (95% CI = 1.0–1.2), 31–45 km aRR = 1.2 (95% CI = 1.1–1.4), 46–90 km aRR 1.0 (95% CI = 0.9–1.1). *P*-values not reported.	Distance to maternity unit: 0–15 km (reference) = 16–30, 31–45, 46–90 km	Yes	Maternal age, sex of newborn, gestational age, high-risk pregnancy, deprivation index, level of urbanisation, unexpected out-of-hospital birth	Distance did not influence neonatal mortality, stillbirth or polycythaemia. Only neonatal hospitalisation increased modestly with farther distance.	Yes (15 Km)
Featherstone *et al.*, 2016 [61]	NMR	aOR = 1.19 (95% CI = 0.86–1.66) for 30–59 min *vs.* <30 min; aOR = 0.83 (95% CI = 0.56–1.22) for ≥60 min *vs.* <30 min. *P*-values not reported but CIs include 1.	Travel time to hospital: <30 min (reference) = 30–59, ≥60 min	Yes	Maternal age, race, previous live births, chronic and gestational diabetes, chronic and gestational hypertension, smoking status, gestational age, infant sex, NICU admission, delivery in level III hospital	In both crude and adjusted analyses, no significant associations were found between travel time and neonatal death among VLBW infants.	Yes (30 min & 1 Hr) home to facility
Lee *et al.*, 2022 [62]	Perinatal mortality	Perinatal mortality rate 2.97 per 1000 births in vulnerable *vs.* 2.92 per 1000 in invulnerable areas, *P* = 0.789.	Accessibility class: invulnerable (reference) = vulnerable PMCSA access	No	None (descriptive comparison only)	Perinatal mortality was slightly higher in vulnerable areas, but the difference was not significant.	
Paranjothy *et al.*, 2014 [63]	Stillbirth and NMR	Every 15-minute increase in travel time. All births: intrapartum stillbirth cOR = 1.29 (95% CI = 1.14–1.47), aOR = 1.13 (95% CI = 0.98–1.30); early neonatal death cOR = 1.37 (95% CI = 1.31–1.45), aOR = 1.13 (95% CI = 1.07–1.20); late neonatal death cOR = 1.33 (95% CI = 1.23–1.44), aOR = 1.15 (95% CI = 1.05–1.26); combined stillbirth + neonatal cOR = 1.36 (95% CI = 1.30–1.41), aOR = 1.15 (95% CI = 1.09–1.20). Term births: intrapartum stillbirth aOR = 1.36 (95% CI = 1.17–1.59); late neonatal death aOR = 1.34 (95% CI = 1.13–1.59); other outcomes mixed; individual *P*-values not reported but CIs often exclude 1.	Travel time to hospital: <15 min (reference) = effect per additional 15 min	Yes	Maternal age, parity, gestational age, infant gender, deprivation index (Townsend), urban or rural residence	Longer travel time to hospital increased the risk of intrapartum stillbirth and neonatal mortality, with associations strongest for intrapartum stillbirth and late neonatal mortality. Effects persisted after adjustment.	
Patel *et al.*, 2022 [64]	NMR (30-day infant survival)	Neonatal mortality <5 miles 10.5% *vs.* ≥5 miles 7.7%, *P* = 0.34; 30-day survival <5 miles 95.5% *vs.* ≥5 miles 97.4%, *P* = 0.60. No OR or HR reported.	Distance from birth location: <5 miles (reference) = ≥5 miles	No (no significant association; multivariable analyses also non-significant)	Not applicable	There was no significant difference in neonatal survival to hospital discharge or 30-day transplant-free survival based on distance from birth location in univariate or multivariable analysis.	
Pilkington *et al.*, 2014 [65]	Stillbirth and NMR	Stillbirth crude RR *vs.* 0–4 km: 5–14 km 0.87; 15–29 km 0.85; 30–44 km 0.85; ≥45 km 0.95, *P* < 0.01. Adjusted RR: 5–14 km 0.99; 15–29 km 1.01; 30–44 km 1.00; ≥45 km 1.08, *P* = 0.51. Neonatal mortality crude RR *vs.* 0–4 km: 5–14 km 0.79; 15–29 km 0.81; 30–44 km 0.77; ≥45 km 0.80, *P* < 0.01. Adjusted RR: 5–14 km 0.91; 15–29 km 0.94; 30–44 km 0.90; ≥45 km 0.96, *P* = 0.01.	Distance to closest maternity unit: 0–4 km (reference) = 5–14, 15–29, 30–44, ≥45 km	Yes	Municipal unemployment, percentage foreign-born residents, percentage single-parent households, maternal age, multiplicity and other socio-demographic characteristics	After adjusting for socio-demographic risk factors, distance did not increase stillbirth or neonatal mortality. Higher mortality was instead linked to urban disadvantage, with women living closer to units often representing higher-risk urban groups.	
Purkey *et al.*, 2022 [66]	Neonatal mortality (0–28 days) and post-neonatal mortality (29–365 days)	aHR = 0.92 (95% CI = 0.62–1.37) for lived close (<11 miles) and transferred (0–28 days); aHR = 1.12 (95% CI = 0.62–2.00) for lived close and transferred (7–28 days); aHR = 1.77 (95% CI = 1.17–2.67) for lived close and not transferred (0–28 days); aHR = 0.97 (95% CI = 0.45–2.07) for lived close and not transferred (8–28 days). Reference group lived far (≥11 miles), not transferred. *P*-values not reported but CI for 1.77 excludes 1.	Distance and transfer status: lived far (≥11 miles), not transferred (reference) = lived close (<11 miles), transferred or not transferred	Yes	Maternal age, race and ethnicity, education, payer, neighborhood poverty, infant sex, transfer status, day of life of arrival	Compared with the lived far/not transferred group, neonatal mortality was higher among the lived close/not transferred group (HR 1.77) but similar for the lived close/transferred group (HR 0.92).	
Singh *et al.*, 2021 [67]	Neonatal survival (24-hour and 1.5-month outcomes)	Median distance survivors 130 km (IQR = 8.7–308.2) *vs.* non-survivors 145 km (IQR = 63.5–447.4); mean distance survivors 329 km *vs.* non-survivors 208 km. Helicopter: mean 380 *vs.* 237 km, median 229 *vs.* 248 km, IQR = 72–447 *vs.* 29–388. Ground: mean 149 *vs.* 6 km, median 54 km *vs.* not reported, IQR = 43–158 *vs.* not reported. No *P*-values reported.	Travel distance (continuous km), compared descriptively between survivors and non-survivors; no formal reference threshold	No (descriptive)	Not applicable	The mean distance travelled was longer for survivors than non-survivors. Helicopter transports covered substantially longer distances than ground transports. No statistical tests or adjusted estimates were provided.	
Swartz *et al.*, 2017 [68]	NMR (30-day survival)	No significant difference in mortality between 11–90 miles *vs.* >90 miles. ROC AUC = 0.5, *P* = 0.9, indicating no discrimination by distance.	Distance group 11–90 miles vs >90 miles; reference not explicitly defined but groups compared	Unclear / not reported	Not applicable	Transfer distance had no impact on 30-day survival. ROC analysis confirmed that distance did not predict outcomes (AUC 0.5).	
**Multi-country studies**							
Karra *et al.*, 2017 [69]	NMR	aORs *vs.* <1 km: 1–1.9 km 1.077 (95% CI = 0.927–1.251), 2–2.9 km 1.163 (95% CI = 1.020–1.327), 3–4.9 km 1.250 (95% CI = 1.087–1.439), 5–9.9 km 1.191 (95% CI = 1.042–1.363), >10 km 1.266 (95% CI = 1.108–1.445). Individual *P*-values not reported; CIs from 2–2.9 km onwards exclude 1.	Distance from facility: <1 km (reference) = 1–1.9, 2–2.9, 3–4.9, 5–9.9, >10 km	Yes	Length of time from child’s birth to survey date, sex of child, single vs multiple birth, mother-, child- and cluster-level controls	Children living ≥2–5 km from a facility had between about 7.7% and 25% higher odds of neonatal death than those <1 km, with odds increasing progressively with distance in a large pooled dataset (n = 125,167).	

aHR – adjusted hazard ratio, aOR – adjusted odds ratio, aRR – adjusted risk ratio, ANC – antenatal care, AUC – area under the receiver operating characteristic curve, cOR / COR – crude odds ratio, CI – confidence interval, CEmONC – comprehensive emergency obstetric and newborn care, EmONC – emergency obstetric and newborn care, FTHG – Federal Teaching Hospital Gombe, HR – hazard ratio, NMR – neonatal mortality rate, NICU – neonatal intensive care unit, OR – odds ratio, PHC – primary healthcare, PMCSA – Perinatal Medical Care Service Area, RR – risk ratio, SB – stillbirth, SD – standard deviation, SMD – standardized mean difference, VLBW – very low birth weight, *β* – regression coefficient

We used several methods to define spatial accessibility, ranging from simple to robust measures (**Table 1**). We computed the majority of proximity metrics using straight-line (Euclidean) distances. This is the simplest distance-based proximity metric and assumes that travel occurs in a straight line; it ignores travel facilitators and barriers (land use, road network and elevation) and does not account for the role of travel mode and speed. The second most common approach in the 37 studies relied on modelling travel time/distance routes, which requires well-defined data sets of roads and paths, often unavailable in LMICs, especially in rural areas. The third approach used in the studies was self-reported proximity. Though travel time is usually the preferred measure of proximity as it accounts for mode of travel and transport speed, most included studies focused on travel distances.

### Studies from LMICs

Twenty-six studies were conducted in LMICs and 19 in sub-Saharan Africa [33–57,69]. Eleven assessed the relationship between proximity and perinatal outcomes using household data [36–40,42,46–48,55,69]. Of the ‘facility-based’ studies (n = 15) that examined a specific patient population, four studies reported on a cohort of admitted newborns [35,41,43,53]. Two studies specified that the cohort was inborn [41,43], one included in- and out-born infants [35], and one looked at newborns admitted for surgery [53]. Of the studies that examined outcomes in patients who were transported to healthcare, six included only outborn infants [33,44,49,50,52,57] and five looked only at inborn infants with a maternal transfer [34,45,51,54,56].

Fifteen studies described the travel endpoint as a hospital, 12 were described as regional or tertiary care facilities, and three as district hospitals [34,45,54]. No studies described newborn services at the origin or travel endpoint by the WHO/UNICEF levels of care. Seven did not include information on care during transport. When transport was mentioned, vehicle type varied from no medical transport to all transfers by ambulance. Descriptions of transport conditions and en route care were sparse, with most reporting only basic monitoring or no clinical support during transfer.

Of the 26 LMIC studies, 25 showed that longer travel time (at any threshold) or distance (at any threshold) was associated with worse perinatal outcomes. Where crude mortality rates were reported, they were consistently higher with increasing distance from hospitals (**Table 3**). One LMIC study could not identify a statistically significant relationship between proximity and a perinatal outcome [40]. Of those that adjusted for confounding (n = 20), three lost statistical significance and 17 maintained it. Overall, the narrative evidence across LMICs demonstrated a consistent, inverse relationship between proximity to hospitals and neonatal survival.

**Table 3:** Available mortality data by travel time band in included studies

Study	Mortality measure	Journey type	Proportion transported by ambulance, %	Deaths/1000 live births by travel time band, n							
**<30 minutes**	**>30 minutes**	**<1-hour**	**>1-hour**	**<2 hours**	**>2 hours**	**<3 hours**	**>3 hours**
Yeshaneh *et al.*, 2021 [57]	Neonatal	Interfacility	74	79	335	190	350	236	407		
Singh *et al.*, 2021 [67]	Neonatal	Interfacility	97					280	500		
Van Duinen *et al.*, 2020 [54]	Perinatal	Home to hospital	42	140	277	175	300	193	308		
Viswanath *et al.*, 2015 [55]	Perinatal	Any	Unspecified					210	420		
Tayler-Smith *et al.*, 2013 [51]	Early neonatal	Interfacility	100							90	210

GIS – geospatial information systems, IQR – interquartile range, km – kilometres, mins – minutes

### Studies from HICs

Eleven studies that were conducted in HICs [58–68] used vital records, hospital discharge data, or disease-specific databases. Six HIC studies assessed the relationship in patients with complications (twin-twin transfusion, very low birthweight, and cardio-pulmonary conditions) [58,61,64,66–68]. All HIC studies specified hospital care, usually at the tertiary level, as the travel endpoint. As in LMIC studies, no HIC studies described origin or destination facilities by the WHO/UNICEF levels of care. Journey origin varied; seven examined home-to-hospital journeys [59–63,65,66] and four studied interfacility transfers [58,64,67,68]. Two studies detailed the care in transport, both describing advanced dedicated paediatric transport [67,68].

Two HIC studies showed a positive relationship between travel time or distance and perinatal mortality [63,66]. These studies adjusted for confounding and reported a persistent relationship. Three studies documented an increase in morbidity or out-of-hospital birth, but no statistically significant relationship with mortality [59–61]. One study from France only showed a distance-mortality relationship for babies born outside the hospital [65]. Five HIC studies, four of which looked at hospital-to-tertiary care transfers, showed no relationship between travel time or distance and perinatal outcomes [58,62,64,67,68].

### Quantitative synthesis (pooled estimates)

We applied a random-effects model using random-effects meta-analysis (REML) estimation; however, as between-study heterogeneity was minimal, the results were nearly identical to those of a fixed-effects model. Because only a subset of studies used comparable exposure thresholds and outcome definitions, pooled estimates are presented for these subsets rather than all included studies.

**Figure 2:**
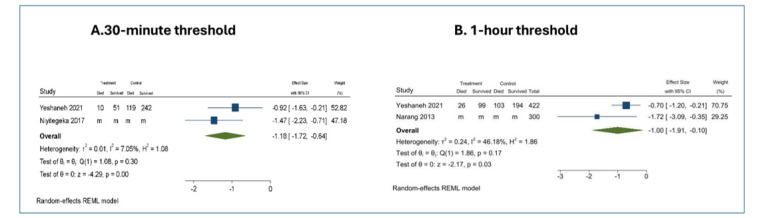
Association between interfacility travel time and perinatal mortality. Forest plots show log odds ratios (log ORs) and 95% CIs from random-effects REML meta-analyses comparing perinatal mortality across interfacility travel time thresholds of 30 minutes (**Panel A**) and 60 minutes (**Panel B**). “m” indicates that individual-level cell counts (deaths/survivals) were missing or not reported in the original study. Effect estimates for these studies were included based on published summary statistics.

Three studies (n = 1163 transfers) estimated effect sizes for perinatal survival in relation to interfacility travel time between lower- and higher-level facilities. An interfacility travel time of less than one hour (compared to more than one hour) was associated with a nearly 3-fold increase in the odds of survival (OR = 2.72; 95% CI = 1.10–6.73) with a further increase in survival when travel times were within 30 minutes (OR = 3.25; 95% CI = 1.90–5.57) (**Figure 2**). The available studies did not support a separate analysis of transfers originating from home to Level II or higher facilities.

**Figure 3:**
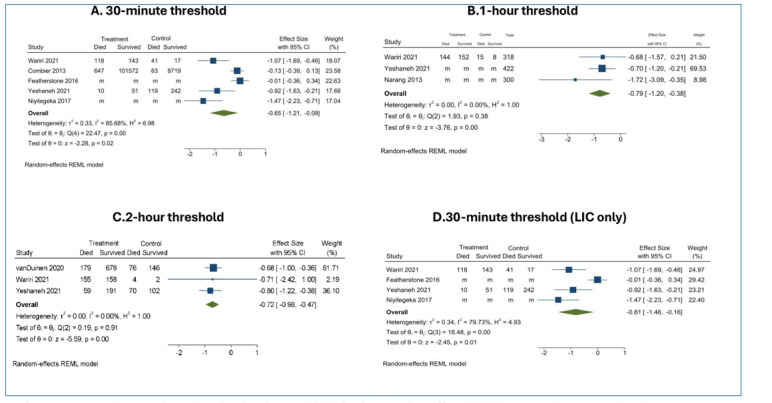
Association between travel time from home or primary-level facility to referral-level facility and perinatal mortality across different time cut-offs. Forest plots show log odds ratios (log ORs) and 95% CIs from random-effects REML meta-analyses evaluating the association between longer travel time from home or a primary-level facility to a referral-level facility and perinatal mortality. Three travel time thresholds were examined: <30 *vs.* ≥30 minutes (**Panel A**), <60 *vs.* ≥60 minutes (**Panel B**), and <120 *vs.* ≥120 minutes (**Panel C**). **Panel D** presents a sensitivity analysis restricted to studies conducted in low-income countries (LIC) using the 30-minute cut-off. “m” indicates that individual-level cell counts (Deaths/survival) were missing or not reported in the original study. Effect estimates for these studies were included based on published summary statistics.

In a pooled analysis (n = 115 592) from any location (home or a lower-level facility) to hospitals (Level II or above), shorter travel times were again strongly associated with improved neonatal survival at all thresholds, with the highest mortality rates observed among neonates with the longest travel times. In the random-effects model, survival odds were significantly higher at each travel time threshold (OR = 2.06; 95% CI = 1.60–2.65 for two hours; OR = 2.20; 95% CI = 1.46–3.33 for one hour; OR = 1.92; 95% CI = 1.10–3.34 for 30 minutes) (**Figure 3**).

We completed a subgroup analysis for the 30-minute threshold in LMICs, as one large study from a high-income setting analysed this exposure. It showed a slightly higher survival benefit at this threshold (OR = 2.25; 95% CI = 1.18–4.32) (**Figure 3**). We interpreted these pooled findings within the broader narrative synthesis to contextualise variation in study design and data completeness. We did not conduct formal tests for publication bias due to the small number of included studies. We descriptively reported heterogeneity statistics (*I*^2^) but did not interpret them as definitive, given the limited number of pooled observations. Due to insufficient data, we were unable to conduct additional subgroup analyses as prespecified in the protocol.

### Risk of bias assessment

Using the NOS, we rated 12 studies as having low, 23 as having moderate, and two as having high risk of bias (Figure S2 in **Online Supplementary Document**). We rated all three case-control studies as having low risk of bias. Six of 12 cohort studies had a low, five had a moderate, and one had a high risk of bias. Among the 22 cross-sectional studies, three had a low, 18 had a moderate, and one had ahigh risk of bias. Cross-sectional studies were more likely to be at moderate or high risk of bias, primarily due to limitations in exposure assessment and confounding adjustment. We considered the study quality when interpreting the consistency rather than the magnitude of associations across studies.

## DISCUSSION

Travel time to hospitals for SSNs was strongly associated with perinatal morbidity and mortality. Across both the narrative synthesis and exploratory pooled estimates, shorter journeys were consistently associated with higher survival. Shorter travel time from any location to hospitals doubled the odds of survival at 30 minutes, one hour, and two hours. For interfacility transfers, the odds of survival more than tripled at a 30-minute threshold. These findings should be interpreted as illustrative rather than inferential, emphasising direction and consistency rather than precise quantitative prediction. Nevertheless, they suggest that the safest place to be born is within 30 minutes of a Level II newborn nursery and are consistent with our best understanding of the physiology of intrapartum complications [15–18]. Though most births are uncomplicated, and most infants require little clinical support beyond essential newborn care, when complications do arise, they demand immediate and often highly skilled attention from clinicians in hospitals. For babies born too soon or with low birthweight, that need is magnified.

The association between our exposure and mortality was weaker in high-income settings but consistent with the mixed literature on the impact of obstetric unit closures and increased travel time from home to birthing facility. While some studies conclude that birth outcomes are compromised when a unit closes, others show the opposite and suggest that people travel farther but receive care in larger, higher quality facilities (*i.e.* ‘trading up’), which improves outcomes [70–73]. In most high-income contexts, nearly all women give birth in a hospital [6,74], which limits comparability with low-resource settings. Only one included study mirrored the dominant LMICs model by examining outcomes in women giving birth out-of-hospital and requiring transfer to a hospital [65]. After controlling for socioeconomic confounders, distance to hospital was only associated with neonatal mortality in the sub-population that gave birth out-of-hospital. Our search did not capture literature on birth outcomes in non-hospital birth centres.

The difference in HICs and LMICs studies may be explained by the rarity of mortality in HICs, making it difficult to detect differences. The weaker relationship may also be explained by the difference in quality of care between HICs and LMICs settings. Although approximately 30% of intrapartum complications occur in dyads without any known risk factors [4,75], high-quality prenatal care should be able to detect the majority and direct those patients to the appropriate level of care for birth. Highly skilled providers at birth would then be able to make timely decisions about transfer and stabilise patients appropriately before they leave. Quality of care across the spectrum of maternal and neonatal healthcare in LMIC settings is often low and missing even basic elements of care [76].

The type and quality of transport varied between settings, though detailed descriptions were often lacking in included studies. Available data showed that many small and sick newborns in LMICs are transferred either by their families or by very basic vehicles, often without any personnel beyond the driver. This stands in stark contrast to emergency transport in settings such as the USA, where sick babies may receive specialised neonatal intensive care during the journey. Consequently, ‘travel time’ functions not only as a measure of geography but also as a proxy for the absence of care during transit – an important contextual modifier of its association with mortality. Despite the lack of high-quality transport, most health systems in low-income settings are designed to depend on referral and transport for childbirth [5]. Women without known prenatal risk factors are deemed safe to deliver in non-hospital settings, the assumption being that if complications arise, the mother or baby will be referred to higher-level care [10].

### Policy implications

While effect sizes appeared similar across the tested thresholds, they represent a gradient of risk rather than equivalence. Each reduction in travel time confers an incremental survival benefit, but the magnitude of that benefit will depend on local service readiness and transport conditions. Organising services to ensure births occur within two hours, one hour, or 30 minutes of Level II care will all improve survival, but the absolute benefit increases as travel time shortens. The appropriate threshold for any given setting should therefore reflect national priorities, resource constraints, and tolerance for perinatal mortality risk. Health planners should interpret the pooled estimates as a directional tool to inform prioritisation, not as a prescriptive cutoff.

Policies or guidelines on travel time for SSNs should be integrated with normative guidance on quality of care [77]. We intended to produce evidence for only one of four norms for Level II SSN units: travel time, workforce ratios, space requirements, and number of units per population. Additional reviews are forthcoming. For example, taken out of context, a travel time norm could incentivise expanded access to low-quality facilities, a resource-intensive strategy which is unlikely to improve outcomes. Importantly, an isolated travel time norm using population-level benchmarks without targeted support for vulnerable populations also risks widening existing disparities [78]. Balanced implementation should therefore link geographic access with investments in care quality and equity.

In the short-term, many LMICs may not have adequate resources to provide effective coverage of Level II neonatal care. Even in well-resourced health systems, governments struggle to provide quality hospital services in low-density geographies [70,79]. In these rural settings, low patient volumes become a significant barrier to sustaining quality services and necessitate additional resources for infrastructure, equipment, and skills maintenance [80]. Where quality hospitals are not available or possible, robust monitoring and reporting systems can identify populations at higher risk due to geographic remoteness, offering targeted access interventions to support travel to safer locations before labour begins. Maternity waiting homes, which are challenging to implement but associated with lower perinatal mortality when co-located with a quality hospital, may be particularly important for these settings [5,81–85]. Higher-risk families should also receive counselling during antenatal care about their elevated risk, such that decisions about the place of birth are informed by contextual realities. There is also a need for standardisation of travel-time computation methods and better integration of geospatial modelling with health-system data to generate realistic, policy-relevant access measures [14,22,86].

### Limitations

Although the review included 37 studies, only a subset provided sufficient data for meta-analysis, particularly for interfacility travel time. This limits statistical power and the ability to robustly assess between-study heterogeneity. We did not conduct formal tests for publication bias, such as Egger’s test and funnel plots, as they are unreliable when fewer than 10 studies are available. This raises concerns about potential publication bias, where studies with null or negative findings may be underrepresented. The included studies varied in methodological quality, with many relying on simplified measures of spatial accessibility – most commonly straight-line distances – which tend to underestimate true travel time. Travel time modelling, though more accurate, was limited by incomplete road and path data in many LMICs, especially rural areas. Few studies accounted for facility bypassing, contributing to exposure misclassification and underestimation of travel time. Accordingly, the pooled results were used only to illustrate consistency of direction, not to imply precision.

The initial protocol anticipated a full meta-analysis; however, substantial heterogeneity observed after screening necessitated a deviation toward a narrative synthesis framework, consistent with SWiM guidance [27]. We retained pooled estimates only where definitions were comparable and are intended to complement, not replace, the qualitative synthesis. Heterogeneity in the description of facility newborn services also prevented pooling by the WHO/UNICEF newborn levels of care. Another limitation is the lack of granular data on potential confounding factors. The absence of individual patient-level data limits our ability to fully account for residual confounding in the relationship between proximity and neonatal survival. Moreover, while efforts were made to assess heterogeneity, sensitivity analyses excluding high-risk studies were not performed due to the limited number of studies. This may affect the robustness of pooled estimates, particularly in the presence of methodological inconsistencies across studies.

## CONCLUSIONS

We found that shorter travel time to hospitals is strongly associated with newborn survival. The relationship is prominent in LMIC settings where absolute mortality rates are higher, and the quality of clinical care and need for transport are lower. Though equitable access to Level II neonatal care is essential for SSNs, the accessibility of services must be balanced with the quality of those services to actualise reductions in mortality. Health system leaders who decide to incorporate a travel time norm into guidance should integrate access and quality standards and consider supporting travel before labour begins for populations living remotely from hospitals.

## Additional material


Online Supplementary Document
