# Supplementary material for: Impact of travel time to health facilities on perinatal outcomes: a systematic review with narrative synthesis and meta-analysis
Source: J Glob Health. 2026 May 15;16:04136. doi: 10.7189/jogh.16.04136 (PMC13178054; doi:10.7189/jogh.16.04136)

Supplement to: Roder-DeWan S, Ramadan M, Ouma P, Manu A, Okiro EA, Strobel N, Robert BN, Odipo E, Macharia PM, Danso-Appiah A, Goodman DC, Dube Q, Gupta G, Magge H, Edmond K. Impact of travel time to health facilities on perinatal outcomes: A systematic review with narrative synthesis and meta-analysis. J Glob Health. 2026;16:04136.

Supplementary Materials

Figure S1: Search Strategy

MEDLINE(R) ALL *via Ovid* <1946 to January 09, 2024>

No.	Query
1	(infan$3 or (newborn or new born or newly born) or (neonat$2 or "neo nat$2") or (baby or babies) or perinatal).ti,ab,kw.
2	Perinatal Care/ or Infant Care/
3	Infant/ or Infant, Newborn/
4	1 or 2 or 3
5	Health Services Accessibility/
6	Travel/
7	regional health planning/ or "catchment area (health)"/
8	(travel* adj3 (distance or time)).ti,ab.
9	(access* adj3 (distance or time)).ti,ab.
10	(geograph* adj3 (distance or time)).ti,ab.
11	("distance to" adj3 (hospital? or service? or unit? or ward? or centre? or center?)).ti,ab.
12	("time to" adj3 (hospital? or service? or unit? or ward? or centre? or center?)).ti,ab.
13	(("travel to" or "travel?ing to") adj3 (hospital? or service? or unit? or ward? or centre? or center?)).ti,ab.
14	(("transport to" or "transported to" or "transportation to") adj3 (hospital? or service? or unit? or ward? or centre? or center?)).ti,ab.
15	(("proximity to" or proxim*) adj3 (hospital? or service? or unit? or ward? or centre? or center?)).ti,ab.
16	(("access to" or accessibility) adj3 (hospital? or service? or unit? or ward? or centre? or center?)).ti,ab.
17	(geographi* adj5 (hospital? or service? or unit? or ward? or centre? or center?)).ti,ab.
18	((geographical information system mapping or GIS) adj3 (hospital? or service? or unit? or ward? or centre? or center?)).ti,ab.
19	(access* or travel* or distance* or transport* or transfer* or proximit* or time* or geographi*).ti.
20	(((level 1 or level i or level one or level 2 or level ii or level two or level 3 or level iii or level three) adj5 (service? or hospital? or ward? or unit? or department? or dept? or centre? or center? or care)) and (babies or baby or newborn* or neonat* or infant* or peri-natal or perinatal or postnatal or post-natal)).ti,ab.
21	5 or 6 or 7 or 8 or 9 or 10 or 11 or 12 or 13 or 14 or 15 or 16 or 17 or 18 or 19 or 20
22	(death* or dead or died or mortalit* or still?birth*).ti,ab,kf.
23	Mortality/ or Infant Mortality/
24	Perinatal Mortality/
25	Survival/
26	Stillbirth/
27	22 or 23 or 24 or 25 or 26
28	Maternal Mortality/
29	4 and 21 and 27
30	21 and 28
31	29 or 30
32	limit 31 to yr="2013 -Current"

Official Use Only

Embase Classic+Embase *via Ovid* <1947 to 2024 January 09>

No.	Query
1	(infan$3 or (newborn or new born or newly born) or (neonat$2 or "neo nat$2") or (baby or babies) or perinatal).ti,ab,kw.
2	perinatal period/ or perinatal care/
3	infant/ or baby/ or newborn/
4	1 or 2 or 3
5	health care access/
6	Travel/
7	health care planning/ or catchment area/
8	(travel* adj3 (distance or time)).ti,ab.
9	(access* adj3 (distance or time)).ti,ab.
10	(geograph* adj3 (distance or time)).ti,ab.
11	("distance to" adj3 (hospital? or service? or unit? or ward? or centre? or center?)).ti,ab.
12	("time to" adj3 (hospital? or service? or unit? or ward? or centre? or center?)).ti,ab.
13	(("travel to" or "travel?ing to") adj3 (hospital? or service? or unit? or ward? or centre? or center?)).ti,ab.
14	(("transport to" or "transported to" or "transportation to") adj3 (hospital? or service? or unit? or ward? or centre? or center?)).ti,ab.
15	(("proximity to" or proxim*) adj3 (hospital? or service? or unit? or ward? or centre? or center?)).ti,ab.
16	(("access to" or accessibility) adj3 (hospital? or service? or unit? or ward? or centre? or center?)).ti,ab.
17	(geographi* adj5 (hospital? or service? or unit? or ward? or centre? or center?)).ti,ab.
18	((geographical information system mapping or GIS) adj3 (hospital? or service? or unit? or ward? or centre? or center?)).ti,ab.
19	(access* or travel* or distance* or transport* or transfer* or proximit* or geographi*).ti.
20	(((level 1 or level i or level one or level 2 or level ii or level two or level 3 or level iii or level three) adj5 (service? or hospital? or ward? or unit? or department? or dept? or centre? or center? or care)) and (babies or baby or newborn* or neonat* or infant* or peri-natal or perinatal or postnatal or post-natal)).ti,ab.
21	5 or 6 or 7 or 8 or 9 or 10 or 11 or 12 or 13 or 14 or 15 or 16 or 17 or 18 or 19 or 20
22	(death* or dead or died or mortalit* or surviv* or still?birth*).ti,ab,kf.
23	infant mortality/
24	mortality/ or "mortality risk"/
25	newborn mortality/
26	Survival/
27	stillbirth/
28	22 or 23 or 24 or 25 or 26 or 27
29	maternal mortality/
30	4 and 21 and 28
31	21 and 29
32	30 or 31
34	limit 32 to yr="2013 -Current"

Official Use Only

Cochrane CENTRAL *via Cochrane Library* | Date Run: 11/01/2024 04:34:55

**ID**	**Search**
#1	MeSH descriptor: [Infant] explode all trees
#2	MeSH descriptor: [Perinatal Care] explode all trees
#3	MeSH descriptor: [Infant Care] explode all trees
#4	(infan* or (newborn or new born or newly born) or neonat* or (neo NEXT nat*) or (baby or babies) or perinat* or peri-nat*):ti,ab
#5	{OR #1-#4}
#6	MeSH descriptor: [Health Services Accessibility] explode all trees
#7	MeSH descriptor: [Travel] explode all trees
#8	MeSH descriptor: [Health Systems Plans] explode all trees
#9	(travel* NEAR/3 (distance or time)):ti,ab
#10	(access* NEAR/3 (distance or time)):ti,ab
#11	(geograph* NEAR/3 (distance or time)):ti,ab
#12	("distance to" NEAR/3 (hospital? or service? or unit? or ward? or centre? or center?)):ti,ab
#13	("time to" NEAR/3 (hospital? or service? or unit? or ward? or centre? or center?)):ti,ab
#14	("travel to" NEAR/3 (hospital? or service? or unit? or ward? or centre? or center?)):ti,ab
#15	("traveling to" NEAR/3 (hospital? or service? or unit? or ward? or centre? or center?)):ti,ab
#16	("travelling to" NEAR/3 (hospital? or service? or unit? or ward? or centre? or center?)):ti,ab
#17	("transport to" NEAR/3 (hospital? or service? or unit? or ward? or centre? or center?)):ti,ab
#18	("transported to" NEAR/3 (hospital? or service? or unit? or ward? or centre? or center?)):ti,ab
#19	("proximity to" NEAR/3 (hospital? or service? or unit? or ward? or centre? or center?)):ti,ab
#20	(proxim* NEAR/3 (hospital? or service? or unit? or ward? or centre? or center?)):ti,ab
#21	("access to" NEAR/3 (hospital? or service? or unit? or ward? or centre? or center?)):ti,ab
#22	(geographi* NEAR/3 (hospital? or service? or unit? or ward? or centre? or center?)):ti,ab
#23	((geographical information system mapping or GIS) NEAR/3 (hospital? or service? or unit? or ward? or centre? or center?)):ti,ab
#24	(access* or travel* or distance* or transport* or transfer* or proximit* or time* or geographi*):ti
#25	((("level 1" or "level i" or "level one" or "level 2" or "level ii" or "level two" or "level 3" or "level iii" or "level three") NEAR/5 (service? or hospital? or ward? or unit? or department? or dept? or centre? or center? or care)) and (babies or baby or newborn* or neonat* or infant* or peri-natal or perinatal or postnatal or post-natal)):ti,ab
#26	{OR #6-#25}
#27	(death* or dead or died or mortalit* or surviv* or stillbirth*):ti,ab
#28	MeSH descriptor: [Mortality] explode all trees
#29	MeSH descriptor: [Infant Mortality] explode all trees
#30	MeSH descriptor: [Perinatal Mortality] explode all trees
#31	MeSH descriptor: [Survival] explode all trees
#32	MeSH descriptor: [Stillbirth] this term only
#33	MeSH descriptor: [Maternal Mortality] explode all trees
#34	{OR #27-#32}
#35	#5 AND #26 AND #34
#36	#26 AND #33
#37	#35 OR #36
#37	Trials
#37	Publication date: 2013 –2024

Figure S2: Risk of bias assessment

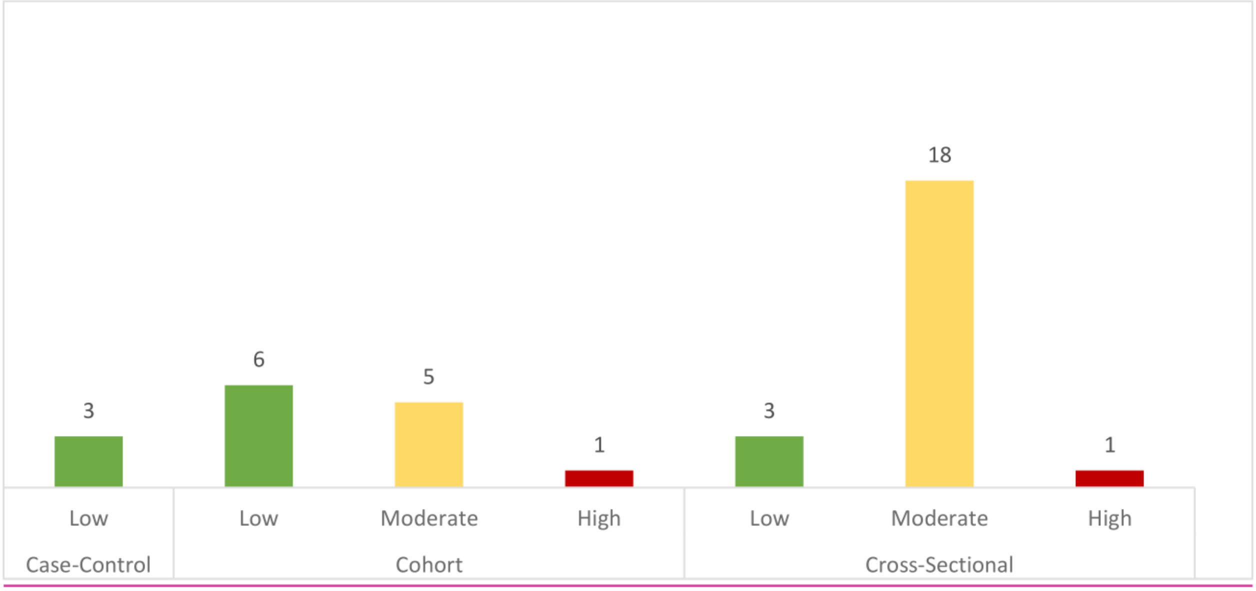

Supplement: Online Supplementary Document [file jogh-16-04136-s001.pdf]
